# Pressure Sensors for Evaluating Hand Grasp and Pinch

**DOI:** 10.3390/s24175768

**Published:** 2024-09-05

**Authors:** Vance Bergeron, Petar Kajganic

**Affiliations:** Laboratoire de Physique, University of Lyon, ENS de Lyon, CNRS, F-69342 Lyon, France; petar.kajganic@ens-lyon.fr

**Keywords:** grasp, pinch, tetraplegia, stroke, Parkinson’s disease, piezoelectric

## Abstract

This study addresses the need for highly sensitive tools to evaluate hand strength, particularly grasp and pinch strength, which are vital for diagnosing and rehabilitating conditions affecting hand function. Current devices like the Jamar dynamometer and Martin Vigorimeter, although reliable, fail to measure extremely low force or pressure values required for individuals with severe hand impairments. This research introduces a novel device, a modified Martin Vigorimeter, utilizing an ultra-soft latex chamber and differential pressure measurement to detect minute pressure changes, thus significantly enhancing sensitivity. The device offers a cost-effective solution, making advanced hand strength evaluation more accessible for clinical and research applications. Future research should validate its accuracy across diverse populations and settings, exploring its broader implications for hand rehabilitation and occupational health.

## 1. Introduction

The human hand usually has five digits: four fingers plus one thumb, these are often referred to collectively as five fingers, wherein the thumb is included as one of the fingers. The hand has 27 bones, not including the Sesamoid bones, the number of which varies among people, and 14 of which are the phalanges (proximal, intermediate, and distal) of the fingers and thumb. The metacarpal bones are the appendicular bones that form the intermediate part of the hand between the phalanges (fingers) and connect the fingers and the carpal bones of the wrist which articulate with the forearm. Each human hand has at least five metacarpals and eight carpal bones.

Fingers contain some of the densest areas of nerve endings in the body and are the richest source of tactile feedback. They also have the greatest positioning capability of the body; thus, the sense of touch is intimately associated with hands. Like other paired organs (e.g., eyes, legs, and feet), each hand is dominantly controlled by the opposing brain hemisphere; therefore, handedness (the preferred hand choice for single-handed activities, such as writing with a pencil) reflects individual brain functioning.

**Figure 1:**
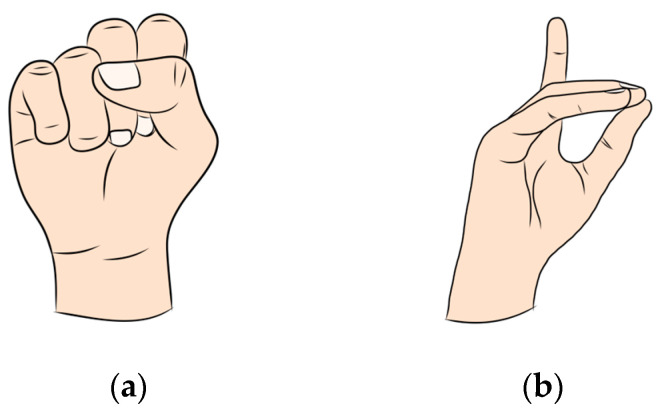
Illustration of (**a**) hand grasp and (**b**) finger pinch.

Grip and pinch strength tests of the hand are fundamental assessments utilized across various health sciences and domains, including the rehabilitation of injured members, ergonomics, sports medicine, and occupational health. These tests quantify the force exerted by the hand muscles during grasping and pinching activities, providing critical insights into hand function. Assessing grip and pinch strength is vital for diagnosing neuromuscular conditions, evaluating hand function, and developing effective solutions for individuals with hand impairments [1]. The particular objective of studying the grip and pinch produced by the human hand is to develop new rehabilitation activities and invent new orthopedic devices (these devices can use electrical stimulation or mechanical movement of the hand/finger muscles or a combination of the two methods) and provide people with limited hand use or non-whatsoever with improved hand dexterity or enable the total use of their hands. This is especially important in the tetraplegic population (complete and incomplete) but can also include stroke victims, Parkinson’s disease sufferers, multiple sclerosis victims, and individuals with other less common diseases. Indeed, amongst the spinal cord injury population, the loss of the use of their hand function is considered the most debilitating feature that limits them from carrying out activities of daily life and improving their quality of life. See Figure 1.

Hand strength is a crucial indicator of upper limb function and is closely linked to the performance of daily living activities. Diminished grip and pinch strength can significantly impact an individual’s ability to carry out tasks requiring fine motor skills and manual dexterity [2]. Thus, reliable and valid assessment tools are essential for measuring hand strength accurately and monitoring changes over time. Ancient Egyptians used basic prosthetic devices, such as the wooden big toe found on a mummy dating back to around 950–710 BC. This prosthetic helped the individual walk and maintain their balance, indirectly aiding hand functions by improving overall mobility. Another historical example can be found in the writings of Hippocrates (c. 460–c. 370 BC), who described splints and other devices used for rehabilitation after injuries. These early devices were designed to help patients regain functionality in their hands and other limbs.

Several factors influence grip and pinch strength, including age, gender, hand dominance, exercise, and the presence of musculoskeletal or neurological disorders [3]. Standardized testing protocols and normative data are necessary to account for these variables and ensure accurate comparisons across different populations. The development of portable and user-friendly dynamometers that use electrical, mechanical, and hydraulic sensors has enhanced the feasibility of conducting these tests in various settings, from clinical environments to field studies [4].

Characterizing hand grasp and pinch strength is essential in evaluating hand function, rehabilitation progress, and the effectiveness of therapeutic interventions. Standardized protocols and norms have been established to ensure consistent and reliable measurements. The **American Society of Hand Therapists (ASHT)** provides comprehensive guidelines for measuring grip and pinch strength using dynamometers and pinch gauges, recommending three types of pinch grips: lateral (key) pinch, tip-to-tip pinch, and three-jaw chuck pinch [2]. The **Jamar dynamometer** is widely recognized for assessing grip strength, with normative data stratified by age and gender [5,6]. In clinical practice, these measurements are typically conducted with the elbow positioned at 90 degrees, the wrist in a neutral position, and the forearm in mid-pronation [7]. These protocols are crucial for diagnosing hand impairments, planning treatments, and assessing outcomes in both clinical and research settings.

Despite the widespread use of grip and pinch strength tests, there is a need for ongoing research to refine testing methodologies, establish comprehensive normative data, and explore the implications of hand strength measurements in different clinical and occupational contexts. For instance, Bohannon et al. highlighted the importance of grip strength as a predictor of overall health outcomes, emphasizing the need for standardized assessment procedures [8]. Moreover, current devices are unable to measure very low force or pressure values.

This paper aims to provide a current overview of the literature on grip and pinch strength testing as well as to introduce a novel device we have developed that makes it possible to evaluate extremely low values of grip and pinch strength (~100 Pa) with the repeatability (precision) ± 10 Pa. This new system is a modification of the Martin Vigorimeter device, which is described below, and will be highly useful as there are currently no grip or pinch evaluation tools that allow for such small values. This will give researchers and clinical practitioners the chance to investigate individuals with severe strength deficiencies and evaluate new tools to assess the strengthening of their muscles and nervous system. This paper seeks to contribute to the understanding of the measurement of hand function evaluation and support the development of evidence-based practices for improving hand strength and overall quality of life for individuals with hand-related impairments.

## 2. Materials and Methods

**Figure 2:**
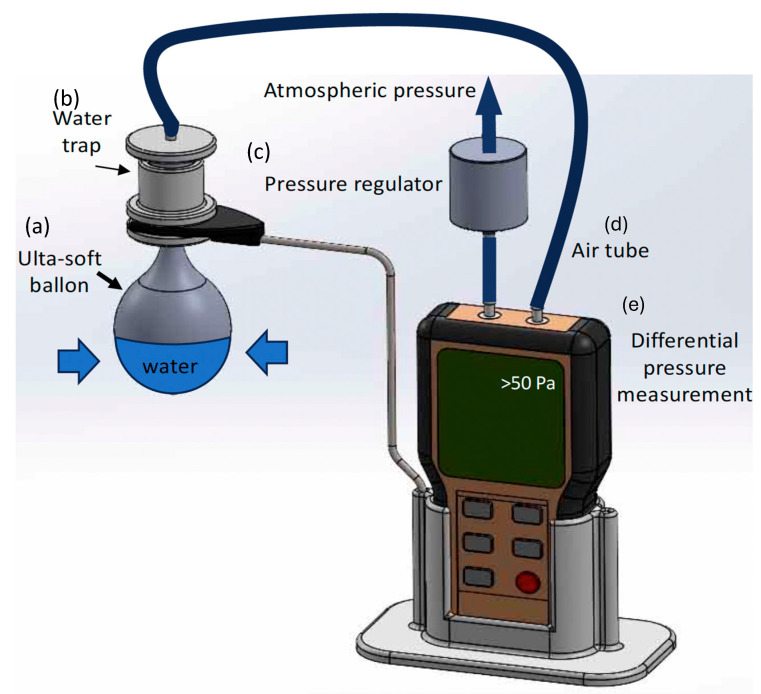
Schematic image of our new differential pressure measurement grasp and pinch dynamometer; (**a**) chamber–ultra-soft water “squeeze bulb” (made of latex with a thickness of approximately 0.08 mm and a stretchiness factor of over 800%) partially filled with water (approx. 30 mL—adjustable for accommodating the hand size of the individual being tested); (**b**) water trap; (**c**) external pressure regimen regulator (optional); (**d**) air tube; (**e**) differential pressure measurement device with ultra-low read out of less than 50 pascals noted, white font on the schematic, repeatability (precision) ± 10 Pa, note: the current limit in the commercial device sold as the Martin Vigorimeter is 1 kPa, which represents a two-order magnitude improvement.

**Figure 3:**
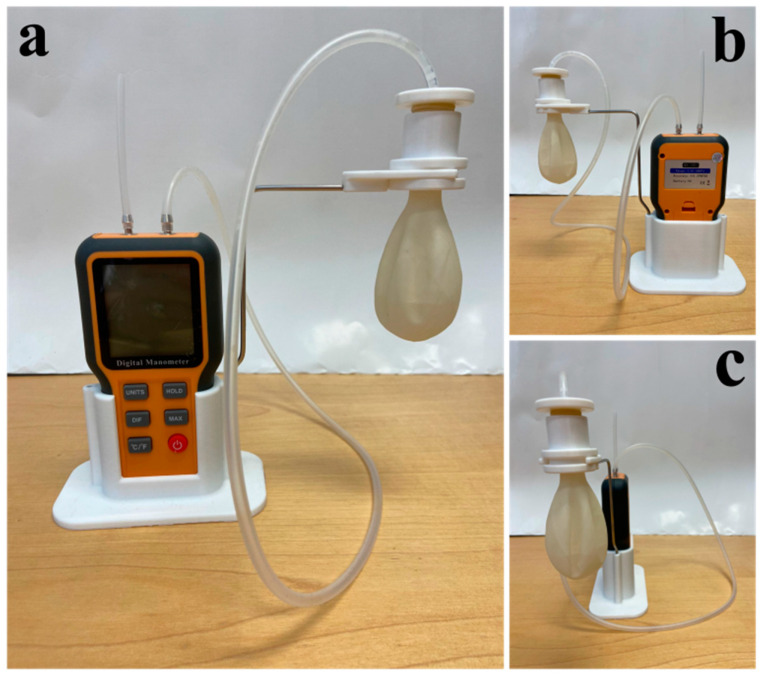
Picture of our new differential pressure measurement dynamometer with approximately 30 mL of water placed in the ultrasoft bulb; (**a**) front view; (**b**) back view; (**c**) side view. The digital differential manometer is the instrument with a yellow color sitting in a 3-D printed white polylactic acid (PLA) plastic stand. The chamber or “squeeze bulb” is hanging from a white plastic 3-D printed water trap retention chamber to protect any water that may overflow through the tube and into the digital instrument. The tube connected to the chamber is 5 mm internal diameter made of silicone plastic. To dangle the chamber, a 3 mm steel bar is used.

**Figure 4:**
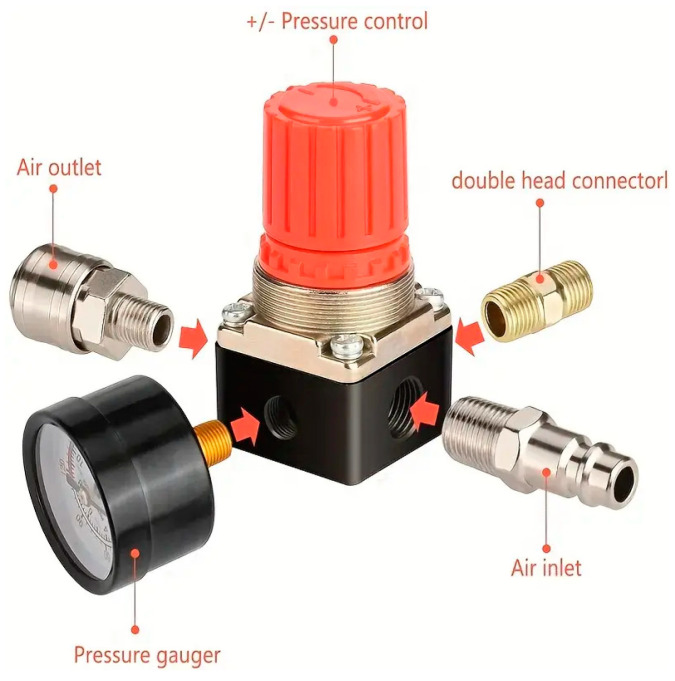
Picture of an “Over Pressure Cut-Off Regulator” (OPCO), a type of pressure regulator used in gas systems to ensure safety by shutting off the flow of gas when the pressure exceeds a predetermined level. The cost of this device on Amazon (Seattle, WA, USA) is EUR 14.39. This is not a necessary item when using our new device for very weak patients (when one knows that the participant does not have enough strength to squeeze the bulb enough to replace the water significantly); therefore, it is shown in Figure 2 schematic as an option but not in the actual image of the instrument shown in Figure 3.

Realizing that there is a need for extremely high sensitivity measurements, we have developed the device schematically portrayed in Figure 2 and photographed in Figure 3. Figure 4 is a photograph of the pressure regulator. This device is a modified Martin Vigorimeter. It uses the same principle of squeezing a sealed flexible container (a latex bulb with 0.08 mm thickness and an 800% stretch factor) and measuring the corresponding pressure change due to volume change, but it is modified to measure extremely low pressures due to the very flexible bulb and sensitive digital differential manometer. Its innovative nature is that it can measure the lowest available reported pinch and grasp pressure values and its low cost with respect to the Martin Vigorimeter (less than EUR 100 (see Section 2) opposed to approximately EUR 518.99, ref. Amazon, for the Martin Vigorimeter).

To realize these features, first, the chamber (“squeeze bulb”) is manufactured out of extremely thin, soft, and easily compliant plastic: 0.08 mm thick latex rubber with an 800% stretch compliance (ex. much less compliant than a standard balloon, Amazon 5 bulb package EUR 8.99, i.e., EUR 1.8 per bulb). Second, the chamber is partially filled (as depicted in Figure 2) with liquid water to expand its size so that a common hand can be maneuvered over the chamber (the chamber size can be changed to accommodate different hand sizes). Once a certain amount of liquid is determined, it should remain constant for each patient so that different experimental results can be easily compared. That said, the pressure exerted by the liquid is always subtracted from the overall measurement so that only the pressures developed by the patient are determined. Moreover, these types of measurements should not be considered strictly quantitative (i.e., one can never guarantee that the patient holds the bulb in the exact position in their hand) but are used to be semi-quantitative, repeatable, and to gauge progress by tracking relative differences. The exact quantity of liquid is not essential. However, it should remain as equivalent as possible for each patient so as not to change the patient’s contact with the squeeze bulb. Furthermore, the pressure due to the water in the bulb is extracted to obtain the experimentally determined pressure exerted by the patient. Although it is easiest to simply use trial and error to determine a convenient bulb size, the precise, mathematical relationship that determines the size can be accomplished by solving a series of differential equations: the shape of a hanging water-filled elastic bulb can be described by a combination of the equilibrium conditions involving forces and the material properties of the bulb. The key forces involved include the weight of the water, the elastic tension in the bulb and the pressure difference across the bulb’s surface. To find the shape, we would solve a system of differential equations that accounts for these forces. For a small element of the elastic squeeze bulb, the fee equilibrium condition can be written as follows:

$$\vec{T\;}\;\hat{n}+∆PA-\vec{W}=0,$$

where

- $\vec{T}$ is the tension force per unit length in the balloon material;
- $\hat{n}$ is the unit normal vector to the surface;
- ∆P is the pressure difference across the balloon surface (internal pressure minus external pressure);
- A is the surface area of the element;
- $\vec{W}$ is the weight of the water inside the element.

Assuming the bulb is thin and elastic, we can apply the **membrane theory**, which gives us a set of differential equations for the balance of forces. In cylindrical coordinates (r,θ,z) for an axisymmetric balloon, we have the following:

$$\frac{d}{dz}\left(T_{z}\right)=\rho gr,$$

$$\frac{1}{r}\frac{d}{dr}\left({rT}_{r}\right)=∆P,$$

where

- T is the tension force per unit length in the balloon material;
- ρ is the density of the fluid;
- g is the gravitational constant;
- ∆P is the pressure difference across the balloon surface (internal pressure minus external pressure).

The tension (T) in the bulb material depends on the strain in the material, which can be modeled using Hooks law for small deformations, and a more complex constitutive relation can be used for large deformations. To solve these differential equations, appropriate boundary conditions are needed: the shape at the top of the bulb (attachment point) and the shape at the bottom of the bulb, where the pressure and tension reach certain specific values. Because the equations are complex when applied to nonlinear material properties, the shape of the bulb is usually obtained via numerical methods, such as the finite element method. The overall equation is not a single, simple formula but rather a system of coupled differential equations that describe the mechanical balance in the bulb requiring numerical computation for specific cases. 

In addition, it is important that the chamber is only partially filled with fluid so that we can take advantage of the compliant air-filled region during stretching. If no liquid was added, any air in the bulb would escape through the differential manometer, and the device could not be used because the bulb would become deflated. Once the bulb chamber is squeezed, the pressure difference caused by the pressure created in the gas portion of the bulb volume will be detected and measured using a highly accurate differential pressure regulator. We use a capacitor-based differential manometer whose operating procedures are fully described at the following website: https://www.mks.com/n/capacitance-manometers (accessed on 2 September 2024). There are many types and brands of differential pressure manometers, and we used the one manufactured by Infurider, which was purchased from Amazon for EUR 45.67. By using this differential pressure device instead of an absolute mechanical pressure gauge, we can use the electronic signal for data storage onto electronic devices, such as a computer or any other digital electronic data collection device. Despite it not being necessary to use over-pressure protection (see Figure 3), particularly for individuals with weak grasp and pinch, it can be a useful low-cost protection feature (EUR < 15 Amazon). As we used our device for very weak persons with incomplete tetraplegia, this feature is not required on our current device. If the measurement technician suspects that the user will significantly overcome the maximum pressure of the manometer, the over-pressure regulator can be useful to protect the electronics in the manometer. Inserting this regulator and squeezing upon the container will not register a value until the pressure resistor is overcome and the final value is registered. 

The white base of the device shown in Figure 2 and the water trap are made of PLA plastic constructed using a 3-D printer (the estimated cost of the plastic used is less than EUR 2). The tube used to carry the airflow was purchased from Amazon at approximately EUR 5. Steel wire, holding the bulb in the air, was handmade in our laboratory with an approximate cost of EUR 1. Adding all of the component costs of the device: differential digital manometer (EUR 45.67), elastic plastic bulb (EUR 1.8), silicone plastic tube (EUR 5), PLA plastic (EUR 2), and steel wire holding brace (EUR 1); the total material cost amounts to EUR 55.47. Three-dimensional printing is performed practically at no cost and the assembly of the device requires only 30 min. If we then include labor construction costs of approximately EUR 20, we find that the entire device cost is approximately EUR 80.

**Figure 5:**
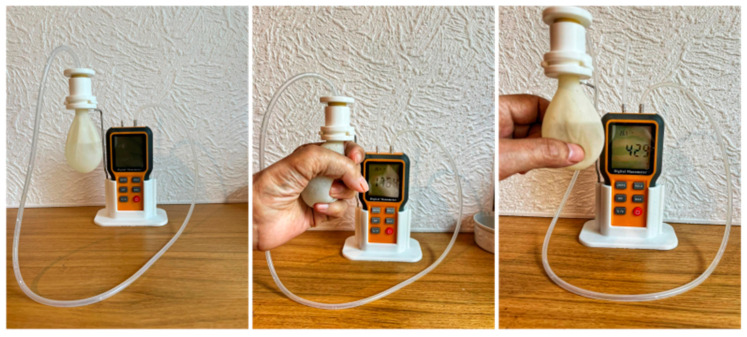
Demonstration of using the new device for grasp and pinch measurements. Reading right to left, the first picture is that of the device without any patient present with the instrument standing alone, while the second image betrays a patient’s hand squeezing the bulb in a typical grasp configuration. Although due to the light glare in this picture the reading is not legible in the figure but we report that the reading on the digital manometer is 1764 Pa is during this grasp action. In the third picture of this Figure the patient is pinching the squeeze bulb and the readout on the digital manometer is 429 pa. We note that this patient also attempted the same grasp and pinch maneuver with standard commercial devices, and in all cases, there was a zero-measurement result.

In order to operate our device, we first note the pressure from the digital manometer with water in the squeeze bulb. We refer to this as the reference pressure. To obtain a measurement, the patient simply squeezes or pinches the bulb and reads the pressure directly from the digital manometer, as demonstrated in Figure 5. In order to obtain the patient’s pressure, the reference pressure is subtracted, only leaving the pressure exerted by the patient.

## 3. Results

**Figure 6:**
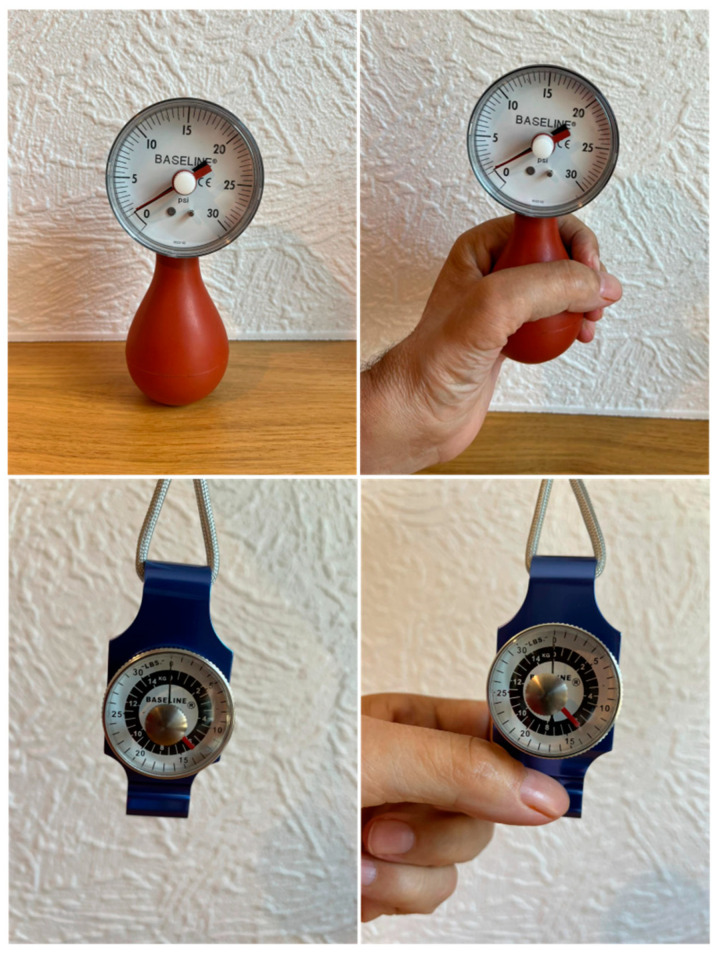
Photographic demonstration of measurement using commercial grasp and pinch devices. The top set of photographs shows a grasp measurement from the tetraplegic author using a commercial Martin Vigorimeter, while the second set of photographs shows a pinch measurement from the same individual using a Baseline pinch instrument (the instrument is described in the Discussion portion below). In both cases, there are no measurable values obtained, and the readings are both zero.

In order to test the feasibility and performance of our new grasp/pinch measuring device (Figure 2 and Figure 3), we developed a proof-of-concept protocol. It should first be noted that all of the commercially available grasp/pinch devices were first tested by the test participant, and they provided a zero-measurement level for every device for both the grasp and pinch measurement (see Figure 6). That said, we know that the individual has a small level of grasp and pinch because he can move all of his fingers independently and lift a plastic 250 mL water bottle horizontally without using the so-called Tenodesis grasp procedure. Tenodesis grasp involves the extension of the wrist (extensor carpi radialis longus and brevis) and can result in two different grasps: a passive whole-hand grip due to finger flexor shortening and a passive lateral grip due to flexor pollicis longus shortening. 

As can be seen in Figure 6, the commercial devices tested provided a 0-level measurement, indicating that the participant had no grasp or pinch in their left hand. However, as can be seen in Figure 5, the same participant was able to produce a measurable grasp of 1764 pascals and a pinch of 429 pascals. We have continued to exercise and perform physical therapy on the left hand of the participant used in this demonstration and we have been able to show that with exercise and physical therapy our new device proves that there has been a positive have strengthened of the grasp and the pinch of the individual, and we will provide a separate publication that demonstrates the use of our new device in a physical therapy routine.

## 4. Discussion

### 4.1. Summary of Available Hand Grasp and Pinch Strength Measuring Devices

Assessing hand grasp and pinch strength is a critical component of evaluating hand function, with numerous commercial devices available for these measurements. The devices vary in design, functionality, and accuracy, catering to different clinical and research needs. This summary review discussion highlights the key commercial devices used to measure hand grasp and pinch strength, based on the current scientific literature.

**Figure 7:**
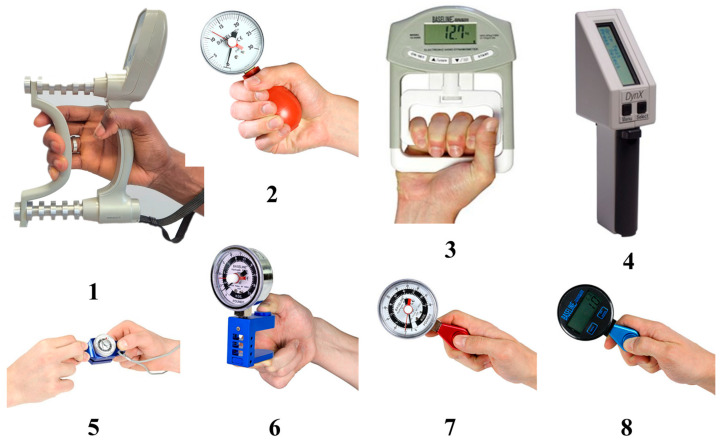
Tools for measuring grip and pinch strengths: (**1**) Jamar dynamometer; (**2**) Martin vigorimeter; (**3**) Smedley dynamometer; (**4**) Dyn X dynamometer; (**5**) mechanical pinch gauge; (**6**) five-position hydraulic pinch gauge; (**7**) hydraulic pinch gauge; and (**8**) digital pinch gauge. *Courtesy of Fabrication Enterprises Inc*., New York, NY, USA [11].

**Figure 8:**
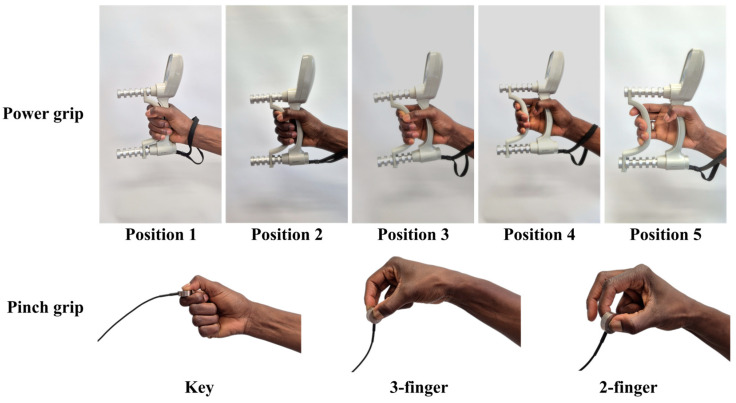
Images of different grip and pinch configurations used to test for grip (hand dynamometer) and pinch (pinch meter) strength during rehabilitation. *Courtesy of Néné Gallé BA, Niamey, Niger*.

Currently, devices used to evaluate grip and pinch strength are based on force or pressure measurements. Unfortunately, most currently available devices do not measure extremely low values (<0.05 kg), which are required for individuals who have a motor handicap due to peripheral or central nervous system damage, severely affecting their hand strength. The minimum force and pressure measurements for evaluating grip and pinch strength in a human hand are typically determined by the sensitivity and resolution of the measuring instruments used, which are in the range rather limited to relatively high values of the force and pressure (0.1 kg). Figure 7 provides an image of commonly used devices used in clinics in healthcare settings; Figure 8 highlights the methodology that is used for the different testing procedures. Further details can be found in the cited references [9,10].

#### 4.1.1. Jamar Hydraulic Hand Dynamometer

The Jamar hydraulic hand dynamometer is considered the gold standard for measuring hand grip strength due to its reliability and validity [1,12]. It features a hydraulic system that measures the force exerted and displays the results on an analog dial (newer systems use a digital readout). It is used due to its ease of use, as well as the fact that numerous studies have shown its high inter- and intra-rater reliability, making it a preferred choice in clinical and research settings [7].

#### 4.1.2. Martin Vigorimeter

The Martin Vigorimeter uses air pressure to measure grip strength and is particularly useful for older patients [13,14] and patients with arthritis [15,16] as it requires less force to compress. It consists of a rubber bulb connected to a pressure gauge, providing an alternative for measuring grip strength in populations with reduced hand function. It has been shown that grip strength measurements are less dependent on hand anthropometry when using the bulb design compared to the Jamar dynamometer design [17,18]. However, the realistic lower limit measurement for this device is above 3 kPa (0.5 PSI) [19].

#### 4.1.3. Smedley Dynamometer

Smedley dynamometers have a different design from hydraulic and pneumatic hand dynamometers. A Smedley dynamometer makes use of a calibrated spring to provide resistance during a grip test. The force gauge is oriented in front of the hand as opposed to above the wrist [20,21]. Similarly, to the Jamar hydraulic hand dynamometer, in newer versions, the results are displayed on a digital screen and can be saved for a limited number of users.

#### 4.1.4. DynX Training Dynamometer

DynX training consists of a series of timed isometric efforts performed by the muscle groups of the hand that can produce grip at levels proportionate to Maximum Voluntary Contraction (MVC) measured for the muscles to be trained. These efforts are interrupted with timed rest periods between each effort. The sustained isometric efforts of the DynX programs (three different programs are available and referred to as; maximum test, endurance test, and rapid exchange test) require only muscle tension. They are purely isometric regimens since there is no movement, and tendons are only stressed and do not move in their sheath. As a result of the lack of motion, no outward work is being carried out. Compliance scores are calculated during the therapy and are totaled during the rest periods. The scores indicate the percent compliance with the prescribed therapy, thereby providing feedback to both the patient and the therapist on the quality of exercise accomplished. Two training regimens, called Fixed Therapy and Stepped Therapy, are provided by DynX. Training compliance data are retained in non-volatile memory along with the parameters of the training modality. These data may be downloaded to a PC for permanent storage prior to initiating another training session. Real-time output of training may be monitored at any time [22].

#### 4.1.5. Mechanical Pinch Gauge

A mechanical pinch gauge uses a mechanical measurement to assess pinch strength. The force exerted on the groove placed between the thumb and the fingers is measured and displayed on an analog gauge. The gauge is usually equipped with a maximum value indicator that remains until reset. A mechanical pinch gauge is used in clinical practice for the assessment of key, three-finger, and two-finger pinch [23]. 

#### 4.1.6. Five-Position Hydraulic Pinch Gauge

A hydraulic 5-level pinch gauge has an adjustable handle, allowing the pinch strength to be assessed at the varied pinch spans (from 2 cm to 6 cm) for accurate and repeatable pinch measurements [24]. Five-position strength test protocols (maximum voluntary exertion, MVE and modified maximum voluntary exertion, MMVE) can now be used for pinch measurements. The easy-to-adjust paddle accommodates any hand size (other pinch gauges only have one pinch width). The pinch width without the paddle is the same width as standard pinch gauges, both mechanical and hydraulic. Five pinch positions permit MVE and MMVE protocols to be used with tip, palmar, and key tests [25]. The measurement results are displayed on a gauge facing toward the practitioner, concealing the reading from the subject.

#### 4.1.7. Hydraulic Pinch Gauge

A hydraulic pinch gauge employs a simple hydraulic system for pinch strength measurements. It can be used for the assessment of key, three-finger, or two-finger pinch. A hydraulic pinch gauge provides repeatability over a long period as it does not rapidly degrade. The main shortcomings of the device are the accuracy, which is typically above 0.4 kg, and the lack of adjustability for different hand shapes and sizes.

#### 4.1.8. Digital Pinch Gauge

The pinch strength measurement using the digitalized pinch dynamometer is reliable within the rater and between raters. It can be used to assess key, three-finger, or two-finger pinch strength, where the results are displayed on a digital screen and saved in the memory of the device. A digital pinch gauge can be used interchangeably with the hydraulic pinch gauge; however, it suffers from similar shortcomings, with a fixed pinch span and a lower limit for measurements of around 0.1 kg [26].

In summary, various commercial devices are available for assessing hand grasp and pinch strength, each with its advantages and specific use cases. Hydraulic dynamometers like the Jamar and Baseline remain the gold standard due to their proven reliability, while digital dynamometers and specialized devices like the Martin Vigorimeter provide additional options for specific populations and settings. Continued research and technological advancements will likely further refine these tools, enhancing their accuracy and utility in clinical practice.

### 4.2. Force Mapping Systems

**Figure 9:**
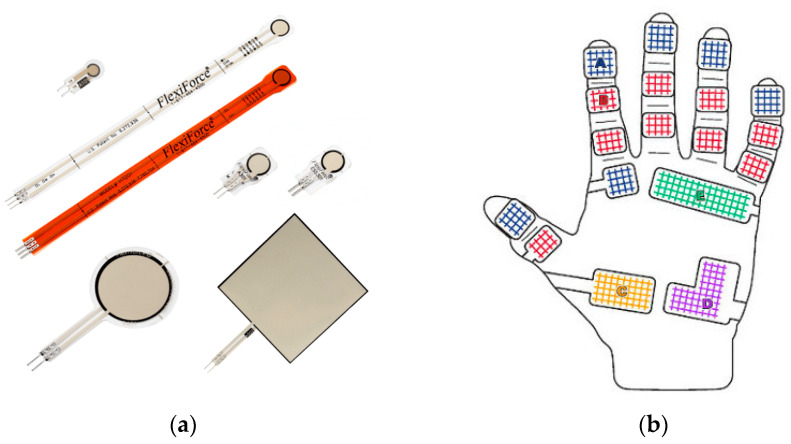
(**a**) Image of typical flexible, piezoelectric elements, for precise images of the individual PCO electric devices. In order for the reader to obtain higher resolution, images and details of these and other PC electric devices used for this application we refer them to the website Tekscan, who provided us with these images [30]; (**b**) schematic image of a glove with piezoelectric elements positioned over critical areas of a glove that fits over a hand to detect force in the corresponding hand positions. On the tip of the index finger there is labeled A (blue) and B (red), electrodes placed on the palm are labeled C (yellow) D (purple) & E (orange). In order for the reader, to obtain higher resolution, images and details of this hand with the PG electric devices placed on his palm, we refer the reader to Tekscan, who provided us with these images [30]. *Courtesy of Tekscan, Norwood, MA, USA*.

Grip and pinch strength evaluations using force-sensing devices that incorporate piezoelectric sensors are crucial for assessing biomechanical function and aiding in research settings (Figure 9). Piezoelectric sensors convert mechanical stress into electrical signals, allowing for the precise measurement of force and pressure applied by the hand. These devices are instrumental in diagnosing conditions, such as carpal tunnel syndrome, monitoring rehabilitation progress, and enhancing ergonomic designs to prevent workplace injuries. Notable devices in this domain include the Biometrics E-LINK Evaluation System [27], which offers comprehensive hand function analysis, and the Noraxon myoFORCE [28], which integrates piezoelectric technology for detailed force measurement. Additionally, the Pliance Hand Diagnostic System by Novel provides dynamic pressure distribution measurement, further extending the capabilities of piezoelectric sensors in hand evaluations [29]. These advanced systems highlight the pivotal role of piezoelectric sensors in delivering accurate and reliable biomechanical assessments.

**Figure 10:**
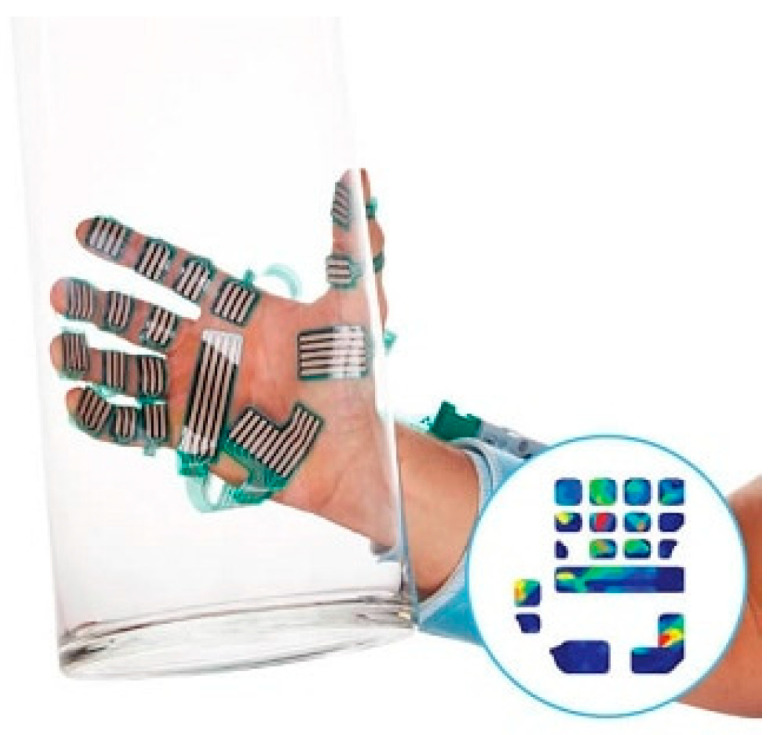
Tekscan Grip™ system for evaluating pressure from grasping objects [31]. The picture is that of a hand holding a glass with piezoelectric electric sensors fixed onto various digits. The circular insert in the bottom right corner depicts color-coded pressure level readouts, blue being the lowest and red the highest pressure measured. For further details concerning these color-coded pressure level readouts we refer you to the origin of this image reproduced from the Tekscan website [30] *Courtesy of Tekscan*.

A commonly used system for measuring pressure distribution and intensity in the hand is the Tekscan Grip™ system [31]. This system uses thin, flexible sensor arrays that can capture detailed pressure data from the hand while performing grip or pinch (Figure 10).

The Tekscan Grip™ system is an advanced tool for evaluating hand grip and pinch strength, leveraging the capabilities of its core component: thin and flexible tactile sensors. These sensors are based on a pressure-sensitive matrix that can measure the force distribution across their surfaces. The sensors comprise a grid of conductive rows and columns, creating an array of intersecting points that register changes in electrical resistance when pressure is applied. As force is applied to the sensor, the contact resistance decreases proportional to the applied force, allowing the system to quantify the force at each point. The grid is made with a high spatial resolution, providing detailed mapping of pressure distribution across the sensor surface. Multiple sensors can be placed on the hand and produce real-time feedback, which is useful for clinical assessment and research applications. The thin and flexible sensors conform to the shape of the hand, providing accurate measurements without significantly altering the natural grip or pinch posture.

The approximate price of the Tekscan Grip™ system can vary based on the specific configuration, included features, and any additional services, such as software licenses, training and support. According to the most recent information, the base price for a Tekscan Grip™ system generally starts around USD 10,000 to USD 15,000. Additional features or upgraded versions of the system can push the price higher, sometimes exceeding USD 20,000.

The Tekscan Grip™ system is highly versatile, finding applications in various fields, such as biomechanics, rehabilitation, ergonomics, and sports science. In clinical settings, it can be used to assess hand function in patients recovering from an injury or surgery, providing detailed data on grip strength and distribution that can guide rehabilitation strategies. In ergonomics, it helps in designing tools and interfaces that optimize human performance and comfort by analyzing how forces are applied during use. Sports scientists use the system to study athletes’ grip techniques, aiming to enhance performance and reduce injury risks.

Other instruments similar to the Tekscan Grip™ system include the F-Scan and the T-Scan systems, which also utilize thin-film pressure sensors. The F-Scan system, for instance, is primarily used for gait analysis, employing sensors that measure plantar pressure distribution to provide insights into foot biomechanics [32,33]. The T-Scan system is designed for dental occlusion analysis, using pressure sensors to map the distribution of bite forces [34].

The transducers used in these systems share a common principle: they are constructed from piezoresistive materials that change resistance under mechanical stress. This allows them to measure and map pressure distribution accurately and in real time. For example, in the F-Scan system, the sensors are embedded in insoles worn by the subject, capturing dynamic pressure patterns as they walk or run. Similarly, the T-Scan’s dental sensors capture the bite force distribution, assisting in diagnosing and treating occlusal disorders.

Devices using similar principles include the XSENSOR X3 PRO V7, which is used for pressure mapping in seating and bedding applications, and the NOVEL Pedal system, which is another tool for dynamic plantar pressure measurement. These devices, like the Tekscan Grip™ system, rely on the piezoresistive properties of their sensors to provide detailed, real-time force and pressure data, proving indispensable in fields requiring precise pressure analysis.

Instruments that incorporate piezoelectric elements, like the Tekscan Grip™ system and similar devices, exemplify the sophisticated application of piezoresistive transducers in various domains, from medical rehabilitation to ergonomic design and sports science. The detailed, quantitative data they provide are crucial for enhancing performance, preventing injury, and improving overall human–machine interactions. It is difficult to use them to determine an overall grasp or pinch measurement for a patient; however, they are fundamental for studying individual muscle groups. These devices are invaluable in clinical settings for evaluating hand function, diagnosing conditions, and planning rehabilitation programs. They provide precise and detailed information about the pressure exerted during grip and pinch activities, even at very low levels.

The human hand, with its intricate anatomy and dense concentration of nerve endings, plays a crucial role in daily activities and overall quality of life. Evaluating hand strength, particularly grip and pinch strength, provides essential insights into hand function, aiding in the diagnosis and rehabilitation of various conditions. This study aimed to address the limitations of current devices in measuring extremely low grip and pinch strength values by introducing a novel, highly sensitive device.

Current commercial devices such as the Jamar dynamometer, Martin Vigorimeter, and Smedley dynamometer, while reliable, have limitations in terms of detecting very low force values. These devices typically cater to higher strength ranges, making them inadequate for individuals with severe hand strength deficiencies, such as those resulting from spinal cord injuries, strokes, or neurological disorders. The newly developed device, a modified Martin Vigorimeter, addresses this gap by using an ultra-soft, compliant latex chamber and a differential pressure measurement system to detect minute pressure changes. This modification allows for the measurement of extremely low values, significantly enhancing the sensitivity range compared to existing devices.

The Tekscan Grip™ system and similar force-mapping systems exemplify the sophisticated application of piezoresistive transducers in hand strength evaluation. These systems provide detailed, real-time data on pressure distribution and force, essential for accurate diagnosis and rehabilitation planning. However, the high cost and complexity of these systems limit their accessibility, particularly in resource-limited settings. The modified Martin Vigorimeter offers a cost-effective alternative, providing high sensitivity without a significant increase in cost, thus potentially increasing accessibility for a broader range of clinical and research applications.

Force sensor devices incorporating various technologies, such as the Biometrics E-LINK Evaluation System and the Noraxon myoFORCE, offer precise force measurement capabilities. These devices are instrumental in diagnosing conditions like carpal tunnel syndrome and monitoring rehabilitation progress. The modified Martin Vigorimeter, while not incorporating piezoelectric technology, provides a comparable level of sensitivity and accuracy in measuring low-force values, demonstrating the potential for simpler, more cost-effective solutions in hand strength evaluation.

The development of this novel device highlights the importance of continued research and innovation in the field of hand strength evaluation. By addressing the limitations of existing devices and enhancing measurement sensitivity, this new tool offers significant potential for improving the assessment and rehabilitation of individuals with severe hand strength deficiencies. Future research should focus on validating the device’s accuracy and reliability across diverse populations and clinical settings, as well as exploring its potential applications in various occupational and therapeutic contexts.

## 5. Conclusions

In conclusion, the human hand’s complexity and its critical role in daily activities necessitate accurate and sensitive tools for evaluating hand strength. Existing devices, while reliable, fall short in measuring extremely low grip and pinch strength values, limiting their utility for individuals with severe hand impairments. The novel device developed in this study addresses this gap by significantly enhancing measurement sensitivity through the use of a highly compliant latex chamber and a differential pressure system.

This innovation provides a cost-effective alternative to more complex and expensive systems, potentially increasing accessibility and utility in various clinical and research settings. The modified Martin Vigorimeter demonstrates that it is possible to achieve high sensitivity and accuracy in hand strength measurement without a significant increase in cost or complexity.

Future research should focus on further validating this device’s performance and exploring its applications across different populations and clinical contexts. By continuing to refine hand strength evaluation tools, we can better diagnose, treat, and rehabilitate individuals with hand impairments, ultimately improving their quality of life.

## Data Availability

No data were collected relevant to the written paper.
